# Transient neurovascular coupling impairment in brain penetrating arterioles of streptozotocin treated mice post recurrent nonsevere hypoglycemia

**DOI:** 10.1016/j.isci.2025.114371

**Published:** 2025-12-09

**Authors:** Irene Fernandez Ugidos, Jennifer Calvo Iglesias, Heather Sendall, Víctor Manuel Calero-Hernandez, Courtney M. Dugas, Andrea Zsombok, Prasad V.G. Katakam, Ricardo Mostany

**Affiliations:** 1 Pharmacology Department, Tulane University, New Orleans, LA, USA; 2 Tulane Brain Institute, Tulane University, New Orleans, LA, USA; 3 Biological Chemistry Program, School of Sciences and Engineering, Tulane University, New Orleans, LA, USA; 4 Physiology Department, Tulane University, New Orleans, LA, USA

**Keywords:** Physiology, Neuroscience

## Abstract

Hypoglycemia is a major complication of insulin therapy, particularly in type 1 diabetes mellitus. During hypoglycemia, the brain initiates vascular adaptations to maintain adequate blood flow. Here, we examined the effects of non-severe recurrent hypoglycemia (RH) on brain vascular diameter and neurovascular coupling (NVC)—the functional mechanism adapting local blood flow to neuronal energetic demand—in a streptozotocin (STZ)-induced hyperglycemia mouse model. We assessed stimulus-induced dilation of penetrating arterioles (PAs) in the somatosensory cortex of awake mice using *in vivo* two-photon microscopy. NVC remains preserved after 8 weeks of sustained hyperglycemia. Non-severe RH episodes, however, cause a transient delay in NVC, which resolves within 24 h. These results suggest that PAs maintain vascular autoregulatory mechanisms despite the metabolic challenges posed by hyperglycemia and RH, highlighting the resilience of brain PAs to systemic glucose fluctuations. We conclude that hyperglycemia per se is not a disruptor of NVC in PAs in the diabetic pathology.

## Highlights

• Neurovascular coupling is preserved under chronic hyperglycemia in brain penetrating arterioles
• Recurrent hypoglycemia transiently delays NVC response in hyperglycemic mice
• Brain penetrating arterioles are resilient to systemic glucose fluctuations

## Graphical abstract

**Figure:**
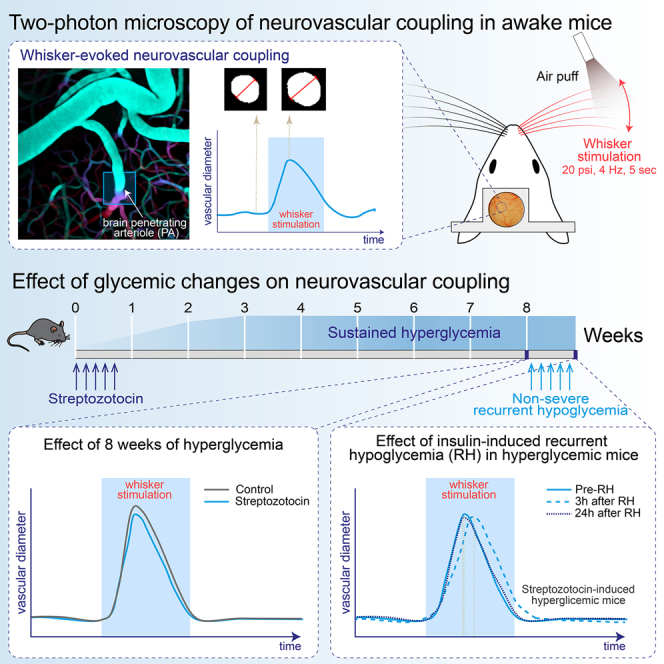

## Introduction

Functional hyperemia, or neurovascular coupling (NVC), refers to the physiological process that ensures that local blood perfusion is adjusted to match neurometabolic demands in the brain.^1^^,^^2^ This mechanism is regulated across different levels of the brain’s vasculature arbor, which can be divided into pial arteries, parenchymal or penetrating arterioles (PAs), and the capillary bed—each playing a distinct role in the NVC response.^3^ Within this vascular system, PAs serve as a critical gateway between the pial arteries and the capillary bed, serving as a key regulatory point for blood flow perfusion across different cortical layers.^4^^,^^5^ Due to their strategic position, PAs act as a bottleneck in the cerebral blood flow, making their dilation essential for increasing blood supply to the capillary bed.^3^^,^^6^^,^^7^

There is significant evidence that diabetes is a strong contributor to vascular damage and NVC impairment.^8^^,^^9^ However, while type 2 diabetes mellitus (T2DM) has been consistently linked to disrupted NVC,^10^^,^^11^ the impact of type 1 diabetes mellitus (T1DM) is less documented. Preclinical studies using hyperglycemic T1DM models have yielded mixed results, some of them reporting NVC impairment,^12^^,^^13^ while others fail to find significant effects.^14^ It is relevant to mention that although both T1DM and T2DM share the hyperglycemic profile, they are distinct diseases with different causes and comorbidities.^15^^,^^16^ One key difference between T1DM and T2DM lies in insulin production and function. T1DM results from the absence of insulin production by the pancreas, whereas T2DM is characterized by insulin resistance in peripheral tissues. Consequently, insulin therapy is a standard treatment for T1DM to regulate blood glucose levels and is also used in some advanced T2DM cases, either as a standalone therapy or in combination with other treatments.^17^ A major clinical complication of insulin therapy is hypoglycemia (blood glucose levels drop below 70 mg/dL), which has been linked to increased risk of dementia^18^^,^^19^ and stroke.^20^ Hypoglycemic episodes occur more frequently in T1DM patients,^21^^,^^22^ although they can also be observed in advanced T2DM cases, but at a lower frequency due to insulin resistance.^19^^,^^23^^,^^24^ While severe hypoglycemia (<40 mg/dL, level 3) is relatively rare, occurring at a rate of 0.1–4.9 episodes per patient per year, mild (54–70 mg/dL, level 1) to moderate (45–54 mg/dL, level 2) hypoglycemia is far more common. T1DM patients experience an average of 60–91 episodes per year.^22^^,^^25^ These figures, based primarily on self-reported data, likely underestimate the actual number of hypoglycemic events due to the development of *hypoglycemia unawareness*, a condition in which autonomic and neuroglycopenic symptoms decrease due to repeated exposure to non-severe hypoglycemic episodes.^17^^,^^26^ Most of the non-severe hypoglycemic episodes are self-managed when symptoms arise, however, recent studies indicate that only 50% of non-severe hypoglycemic episodes are detected by patients^27^ due to the development of hypoglycemic unawareness,^23^ making it a silent but significant risk factor for dementia and cerebrovascular events in diabetic patients.

Hypoglycemia not only increases the risk of cardiovascular disease^28^^,^^29^^,^^30^ but also induces vascular changes that can disrupt NVC.^31^^,^^32^ However, the specific effects of recurrent and unreported hypoglycemic episodes on brain vasculature remain largely unexplored. In this study, we investigated whether alterations in systemic blood glucose levels—specifically sustained hyperglycemia (modeling the persistent hyperglycemia associated with T1DM) combined with repeated episodes of non-severe hypoglycemia (modeling brief insulin-induced hypoglycemic events)—affect functionality of brain PAs. The present longitudinal *in vivo* study shows that hyperglycemia alone does not impair the functionality of the arterial vascular component in the brain. However, non-severe systemic hypoglycemic episodes in sustained hyperglycemic conditions appear to induce transient alterations in NVC, that are subsequently recovered.

## Results

### PAs in the primary somatosensory cortex display stochastic dilation dynamics following whisker stimulation

**Figure 1:**
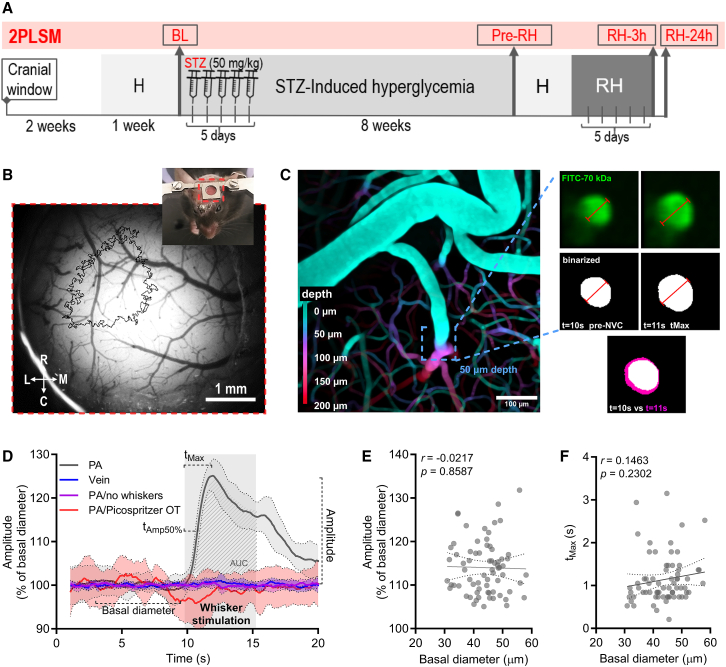
Experimental design (A) Timeline of the study. (B) Cranial window implanted over the parietal cortex of a mouse showing the custom-designed head bar employed (inset). Main picture shows a close-up of the superficial vasculature and the delineated location of the most caudal and medial portion of S1BF obtained by IOS imaging. Scale bar: 1 mm. (C) Representative figure showing a pseudo color depth-coded z stack of the vasculature in S1BF. Expanded images represent cross-section of a PA (top row: raw images; middle row: binarized images) right before the onset of whisker stimulation (left) and at the peak of the stimulation-induced vascular dilation (right). The bottom image shows the overlay of the binarized cross-sections of the PA at pre-NVC (white) and at *t*_Max_ (purple). Scale bar: 100 μm. (D) Experimental proof of principle: NVC is only elicited in PAs and only when whiskers are present and are directly stimulated by air puffs. Solid lines represent the average and the shaded bands represent the SEM of the traces. (E) NVC amplitude and (F) changes in NVC *t*_Max_ as a function of the PA basal diameter show no correlation (Pearson’s *r* correlation) represented as the best linear fit and SD of the values. 2PLSM, two-photon laser scanning microscopy; AUC, area under the curve; BL, baseline; C, caudal; H, habituation; L, lateral; M, medial; NVC, neurovascular coupling; OT, off-target; R, rostral, RH, recurrent hypoglycemia; S1BF, somatosensory barrel field cortex; STZ, streptozotocin; *t*_Amp50%_, time to reach 50% of maximum amplitude; *t*_Max_, time to reach maximum amplitude.

We analyzed the NVC response of PAs at 50 μm deep in the S1BF (Figures 1A–1C). First, we demonstrated that PA dilation is triggered exclusively by external whisker stimulation by measuring NVC responses under various conditions that should not elicit a vascular response, such as whisker stimulation on whisker-trimmed animals or when the picospritzer was out of target (OT) and not aiming at the whiskers (Figure 1D). We also assessed the vein responses to whisker stimulation. We observed that dilation occurs only in PAs, only when whiskers are stimulated by air puffs, and not with spontaneous whisking or in the absence of whiskers. To rule out sampling bias affecting our results, we analyzed the basal dynamics of 70 PAs across the S1BF of 24 mice before diabetes induction. We found that the amplitude and the *t*_Max_ of each PA do not correlate with the basal diameter of the PA (Figures 1E and 1F), indicating that the amplitude and timing of the NVC response is unique to each PA highlighting the need for longitudinal studies when NVC is being analyzed in individual PAs.

### NVC response remains largely unaffected in the PAs of the somatosensory cortex 8 weeks after STZ-induced hyperglycemia

**Figure 2:**
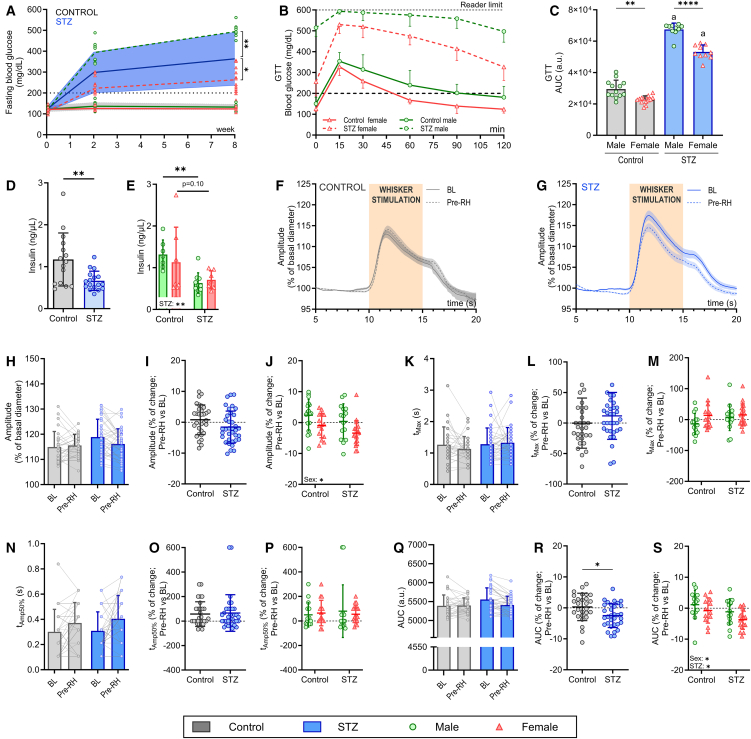
No changes in the NVC response after 8 weeks of sustained hyperglycemia (A) Evolution of fasting blood glucose levels over 8 weeks in control and STZ-treated mice. Blue line with blue shadow represents the average ± SD values of fasting blood glucose in STZ-treated mice (male and female mice combined), while the black line with gray shadow represents the non-induced or control mice. Green and red lines represent males and females, respectively, in both control (solid line) or STZ-treated (dotted line) mice. (B) Blood glucose curves of GTT in males (green) and females (red) of both control (solid line) and STZ-induced hyperglycemic (dotted line) mice. Values represent the average ± SD. (C) AUC computed from GTT traces. Two-way ANOVA followed by Tukey’s post hoc test. Values represent the average ± SD. Insulin levels of control and STZ mice, both averaged (D) and separated by sex (E). Values represent the average ± SD. Population traces of the NVC response in both control (F) and STZ (G) mice at the different time points of the study, represented as averaged population traces ± SEM. NVC changes in amplitude (H–J), *t*_Max_ (K–M), *t*_Amp50%_ (N–P), and AUC (Q–S) in both control and STZ mice *n* = 31 PAs from 10 mice in control and *n* = 36 PAs from 10 mice in STZ; *n* = 6 males and 4 females in both Control and STZ. Values represent the average ± SD. Statistical differences were assessed using two-way ANOVA followed by a Tukey’s post hoc test (A, C, E, J, M, P, S), unpaired Student’s *t* test (D), two-way repeated measures ANOVA followed by a Tukey’s post hoc test (H, K, N, Q), or nested *t* test (I, L, O, R); ∗*p* < 0.05; ∗∗*p* < 0.01; ∗∗∗∗*p* < 0.0001. AUC, area under the curve; GTT, glucose tolerance test; NVC, neurovascular coupling; PA, penetrating arteriole; STZ, streptozotocin; *t*_Amp50%_, time to reach 50% of maximum amplitude; *t*_Max_, time to reach maximum amplitude.

We employed a well-established methodology based on STZ injections to induce sustained hyperglycemia in both male and female mice.^33^ Fasting blood glucose levels increased beyond the hyperglycemic threshold (200 mg/dL) 2 weeks after STZ administration in both sexes (Figure 2A) and further increased with respect to the control group 8 weeks after STZ administration. Notably, we observed a sex-specific response to hyperglycemia development following administration of 50 mg/kg STZ, with male mice exhibiting higher fasting blood glucose levels than females (two-way ANOVA: sex F(1,38) = 46.79, *p* < 0.0001; Tukey’s post hoc test male vs. female at 8 weeks *p* < 0.0001), consistent with previous reports (Figure 2A).^34^^,^^35^ Attempts to equalize hyperglycemia levels between sexes by increasing the STZ dose to 75 mg/kg in females resulted in severe adverse effects, including excessive body weight loss and high mortality rates in the weeks following STZ injections. Despite this increased dose, fasting glucose levels in females never reached those observed in males treated with 50 mg/kg STZ (data not shown). For this reason, we conducted the study using an equal dose of 50 mg/kg STZ for both sexes. GTT and plasma insulin levels confirmed that both male and female STZ-treated mice developed glucose dysregulation, a hallmark of T1DM (Figures 2B–2E). In agreement with previously published studies,^36^^,^^37^ even in control mice, males and females exhibited distinct GTT dynamics, indicating an inherent sexual dimorphism in glucose metabolism (two-way ANOVA: STZ F(1,43) = 730.3, *p* < 0.0001; sex F(1,43) = 69.64, *p* < 0.0001; Tukey’s post hoc test: control male vs. female: *p =* 0.001; Tukey’s post hoc test: STZ male vs. female, *p* < 0.0001) and insulin production. In this sense, plasma insulin levels were significantly reduced in STZ-treated mice compared to controls (Figure 2D; *t* test: control vs. STZ, *p* = 0.0042), displaying sex-dependent differences consistent with those observed in glucose dynamics (Figure 2E; two-way ANOVA: STZ F(1,27) = 10.80, *p =* 0.0028; Tukey’s post hoc test male control vs. STZ, *p =* 0.0057; female control vs. STZ, *p =* 0.1001).

To assess the impact of sustained hyperglycemia on NVC in PAs, we compared whisker stimulation-induced dilation in the same PAs within each animal before hyperglycemia (BL) and 8 weeks post-hyperglycemia induction (Pre-RH; Figures 2F–2S). While dilation amplitude showed a decreasing trend in hyperglycemic conditions, particularly in females, no significant differences were detected (Figures 2H–2J). Temporal dynamics (*t*_Max_ and *t*_Amp50%_) remained unchanged, with no evident sex differences (Figures 2K–2P). However, the AUC of the dilation trace was significantly reduced in STZ-hyperglycemic mice (*t* test: *p =* 0.0107), likely due to the trend toward decreased amplitude, with females primarily driving this effect (Figures 2Q–2S).

### Recurrent hypoglycemia transiently delays the stimulation-dependent vascular dilation of PAs of STZ-treated mice

**Figure 3:**
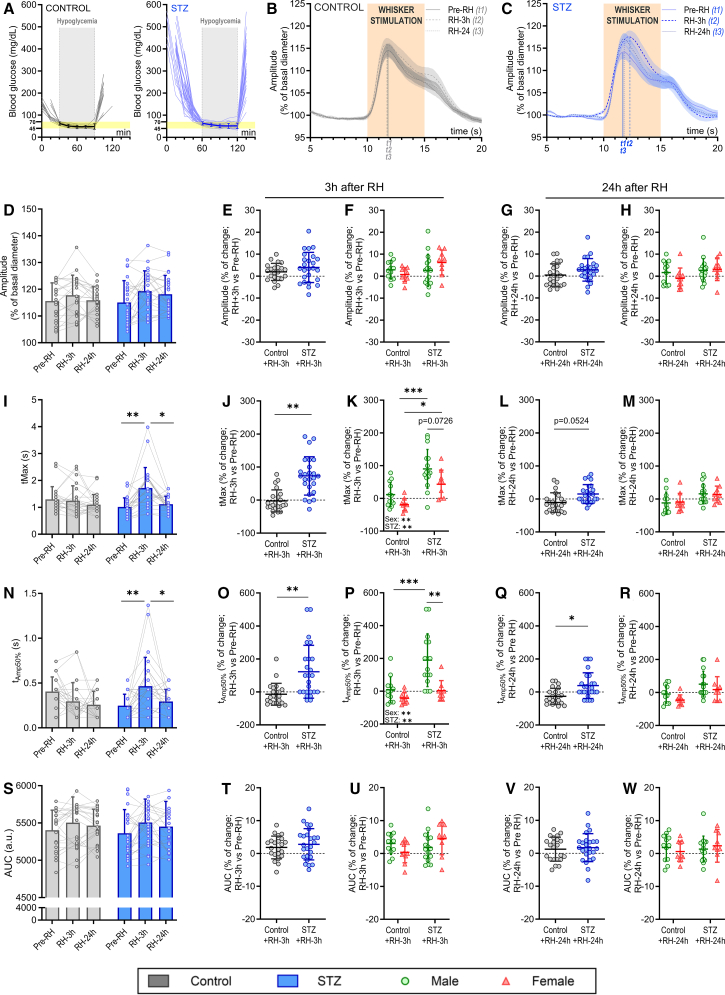
Effect of RH on NVC at 3 and 24 h after the last hypoglycemic episode (A) Individual glucose profiles (thin lines) of both control (left) and STZ-treated (right) mice during recurrent hypoglycemia episodes. The average population blood glucose values during hypoglycemia are represented with thick lines. Population trace of the NVC response before and after RH in (B) control and (C) STZ-treated mice, represented as averaged population traces ± SEM. NVC changes in amplitude (D–H), *t*_Max_ (I–M), *t*_Amp50%_ (N–R), and AUC (S–W) in both control and STZ mice. *n* = 22 PAs from 6 mice in control (*N* = 4 males, 2 females) and *n* = 25 PAs from 8 mice in STZ (*N* = 5 males, 3 females). Values represent the average ± SD. Statistical differences were assessed using two-way repeated measures ANOVA followed by a Tukey’s post hoc test (D, I, N, S), nested Student’s *t* test (E, G, J, L, O, Q, T, V), or two-way ANOVA followed by a Tukey’s post hoc test (F, H, K, M, P, R, U, W); ∗*p* < 0.05; ∗∗*p* < 0.01; ∗∗∗*p* < 0.001. AUC: area under the curve; NVC: neurovascular coupling; PA: penetrating arteriole; RH: recurrent hypoglycemia; t_Amp50%_: time to reach 50% of maximum amplitude; t_Max_: time to reach maximum amplitude.

To assess the impact of non-severe recurrent hypoglycemia (RH) episodes on NVC, we compared the NVC response of individual PAs before RH (Pre-RH) and at 3 h and 24 h post-RH (RH-3h and RH-24) in both control and STZ-treated mice (Figure 3). During each hypoglycemic episode, blood glucose was maintained between 45 and 70 mg/dL for 1 h, followed by a glucose rescue injection if needed, and a subsequent recovery period (Figure 3A). Throughout the hypoglycemia episode, animals remained conscious and active in their cages. Recovery was complete, with no gross differences in behavior from non-hypoglycemic mice and showing levels of blood glucose consistently above 80 mg/dL 3 h immediately after the hypoglycemic episode.

When we analyzed the dilation dynamics in response to whisker stimulation, we found no statistically significant differences in amplitude at 3- or at 24-h post-RH in either control or STZ mice (Figures 3B–3H). However, in STZ mice, NVC response was delayed at 3-h post-RH compared to pre-RH, with a stronger effect in males (Figures 3I–3K; two-way repeated measures ANOVA (I): time point x STZ F(1438,64.71) = 11.85, *p =* 0.0002; Tukey’s post hoc test: STZ pre-RH vs. RH-3h, *p =* 0.0021; RH-3h vs. RH-24h, *p =* 0.012; Nested *t* test (J): control vs. STZ, *p =* 0.0054; two-way ANOVA (K): STZ F(1,43) = 27.88, *p* < 0.0001; sex F(1,43) = 8.423, *p =* 0058; Tukey’s post hoc test: male control vs. STZ, *p =* 0.0002, female control vs. STZ, *p =* 0.02; STZ male vs. female, *p =* 0.0726), indicating an acute RH-induced vascular dysregulation. This delay was largely undetectable at 24 h, though a strong trend persisted in STZ-treated mice (Figures 3L–3M; Nested *t* test (L): 24 h control vs. STZ, *p =* 0.0524). Similarly, the *t*_Amp50%_ followed the same pattern (Figures 3N–3R; two-way repeated measures ANOVA (N): time point x STZ F(1438,64.71) = 9.596, *p =* 0.0007; Tukey’s post hoc test: STZ pre-RH vs. RH-3h, *p =* 0.0061; RH-3h vs. RH-24h, *p =* 0.036; Nested *t* test (O): control vs. STZ, *p =* 0.0015; two-way ANOVA (P): STZ F(1,43) = 12.30, *p =* 0.0007; sex F(1,43) = 13.5, *p =* 0.0011; STZ x sex F(1,43) = 4.372, *p =* 0.0425; Tukey’s post hoc test: male control vs. STZ, *p =* 0.002; STZ male vs. female, *p =* 0.0013), with detectable differences still present at 24-h post-RH in STZ-treated mice (Figure 3Q). Control mice subjected to RH showed no delay in vascular dynamics. The AUC remained unchanged in both groups (Figures 3S–3W), indicating that RH affects only the temporal dynamics of dilation in STZ mice and not its magnitude.

### Basal diameter of PAs is maintained under hyperglycemic and RH conditions

**Figure 4:**
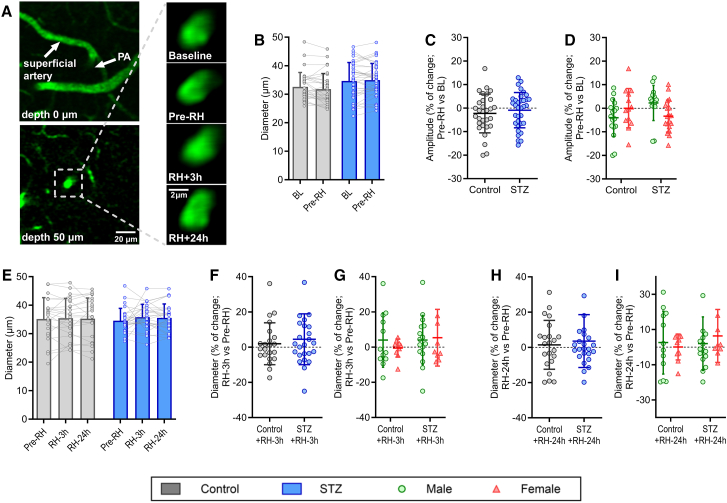
No changes in basal diameter in PAs (A) Representative images of a PA throughout the four imaging time points of the experiment. Scale bars: 20 μm (left, low magnification images) and 2 μm (right, high magnification images). (B−D) Changes in basal diameter of the same PAs in control and diabetic mice between baseline (BL) and after 8 weeks of sustained hyperglycemia (Pre-RH or STZ). (E–I) changes in basal diameter between Pre-RH, 3 h, and 24 h after RH. B–D: *n* = 31 PAs from 10 mice in control and *n* = 36 PAs from 10 mice in STZ, 6 males and 4 females in both control and STZ; E–I: *n* = 22 PAs from 6 mice in control (4 males, 2 females) and *n* = 25 PAs from 8 mice in STZ (5 males, 3 females). Values represent the average ± SD. Statistical differences were assessed using two-way ANOVA followed by a Tukey’s post hoc test (B, D, G, I) or nested *t* test (C, F, H). BL, baseline; NVC, neurovascular coupling; PA, penetrating arteriole; RH, recurrent hypoglycemia.

We analyzed the basal diameter of PAs to ensure that the absence of changes in NVC was not due to alterations in their resting diameter (Figure 4A). No significant differences in basal PA diameter were observed in either hyperglycemic mice (Figures 4B–4D) or after RH (Figures 4E–4I) across any of the analyzed time points or conditions. These findings indicate that PA diameter remains stable under sustained hyperglycemia and following RH.

### Delayed vascular response observed in diabetic mice after RH is driven by hyperglycemic levels rather than insulin dosage

**Figure 5:**
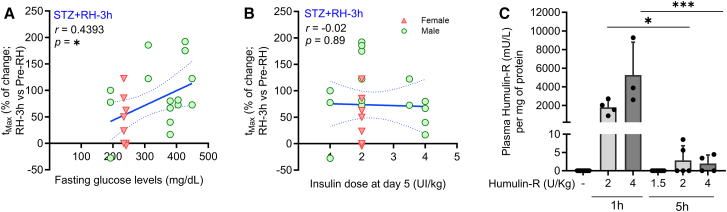
Delayed NVC response in diabetic mice subjected to RH does not correlate with fasting blood glucose levels or with the insulin dose administered (A) Change in *t*_Max_ as a function of the glucose levels. (B) Change in *t*_Max_ as a function of the insulin dose administered on the last day of the RH protocol of STZ+RH mice at 3 h after the last episode of RH. *n* = 28 PAs from 9 mice. (C) Plasma Humulin-R levels in blood at 1 and 5 h after different insulin administration dosages. Pearson’s correlation (A and B) represented as the best linear fit and SD of the values. Statistical differences were assessed using two-way ANOVA followed by a Tukey’s post hoc test (C); values represent the average ± SD. ∗*p* < 0.05; ∗∗∗*p* < 0.001. NVC, neurovascular coupling; RH, recurrent hypoglycemia; *t*_Max_, time to reach maximum amplitude.

Previous studies from our team demonstrated that insulin exerts a direct vasodilatory effect on brain vasculature in *ex vivo* preparations.^38^ While we observed no changes in basal vascular diameter, suggesting that exogenous insulin levels were insufficient to trigger vascular dilation, we analyzed the relationship between fasting glucose levels, insulin dosage, and the *t*_Max_ delay at 3-h post-RH in STZ-treated mice. A significant positive correlation between fasting glucose levels and *t*_Max_ delay (Figure 5A) indicates that hyperglycemia severity influences the RH-induced vascular response delay. However, no correlation was found between the last insulin dose administered and the *t*_Max_ delay in PAs (Figure 5B), suggesting that circulating insulin is unlikely to contribute to the impaired NVC response.

Additionally, serum Humulin-R levels measured 3-h post-RH (approximately 5 h after insulin injection) were nearly undetectable compared to the high levels observed at 1-h post-injection (during hypoglycemic peak; Figure 5C). This further supports the notion that insulin is unlikely to interfere with the delayed NVC response in PAs at this time point.

## Discussion

The present study provides novel insights into the effects of non-severe RH on NVC in PAs of the somatosensory cortex in an STZ-induced hyperglycemic model. Our findings indicate that PAs exhibit a unique resilience to NVC impairments under hyperglycemia and RH conditions, showing only some mild deficits in sustained hyperglycemia, and a transiently delayed vascular response after non-severe RH. This raises the question as to whether PAs remain largely unaffected by NVC modulation and whether their role differs from the roles of the other vascular compartments.

NVC is a compartmentalized process, with distinct contributions at the different levels of the cerebrovascular tree. Pial arteries, PAs, and capillaries each regulate blood flow through specialized mechanisms. Capillaries, for instance, serve as the primary site for spatial blood redistribution, relying on pericytes and endothelial signaling to fine-tune perfusion in response to neural activity.^39^ Pial arteries, on the other hand, integrate upstream regulatory inputs that influence broader hemodynamic changes.^40^ In contrast, PAs function as pressure-regulating conduits, bridging the larger pial arteries with the dense capillary network.^5^ Given their critical role in stabilizing perfusion across cortical layers, we hypothesize that PAs maintain their functional integrity despite glycemic fluctuations.

In this regard, our findings demonstrate that PAs maintain their basal diameter following hyperglycemic induction and RH, indicating that their vascular tone remains stable. These results suggest that PAs possess robust autoregulatory mechanisms that prevent potential dysregulation in NVC under very different glycemic conditions.

In this sense, the assessment of NVC using two-photon imaging in awake mice is specifically designed to understand the contribution of PAs to this functional response. While assessing NVC employing other techniques, such as laser speckle or laser doppler, may identify changes in regional blood flow induced by either hyperglycemia or RH, those approaches inform about the global distribution of blood flow, which is mainly driven by capillary function.^41^^,^^42^ Indeed, capillaries may be more sensitive to metabolic changes due to their reliance on metabolic and endothelial signaling.^43^ Under metabolic stress conditions, such as hyper- and hypoglycemia, capillary function may be more susceptible to impairment, a possibility that is worth exploring in future studies. In contrast, the ability of PAs to maintain stable vascular tone and preserve their function after eight weeks of sustained hyperglycemia or after mild-to-moderate RH episode suggests that this vascular compartment operates independently of systemic metabolic fluctuations, ensuring consistent cerebral perfusion even in altered glycemic states, and aligning with the theory of vascular compartmentalization in the brain.^3^

### Transient delay on PA’s dilation in STZ-hyperglycemic mice

Although PAs remained largely unaffected in terms of dilation amplitude, we observed a delayed NVC response in STZ-treated mice after RH. This indicates that while PAs preserve their dilation capacity, the temporal component of their response to stimulation can be transiently disrupted when sustained hyperglycemia and RH occur simultaneously. In this sense, the effects of hypoglycemia on brain vasculature have been documented in the literature, showing that during hypoglycemic episodes, vessel tone is increased in an astrocyte-dependent manner.^44^ This astrocyte-dependent dilation takes effect when blood glucose levels drop below 70 mg/dL, indicating that even mild to moderate hypoglycemia impacts brain vascular regulation. Despite evidence of increased vascular tone during the hypoglycemic episodes, NVC remains preserved in control mice, relying on the adaptations of both neuronal activity and vascular dilation to low glucose conditions.^45^ Whether hypoglycemia impact lingers after recovering from the hypoglycemic episode has not been explored so far, nor in control, STZ-treated, or other model of diabetic mice. We found that 3 h after RH, when euglycemic levels have been restored, NVC dynamics are unaltered in control mice. Interestingly, in STZ mice, NVC response is delayed without affecting the amplitude nor the basal vascular tone. These results show that STZ-induced hyperglycemia makes PAs more vulnerable to RH episodes, delaying the recovery of their homeostatic state. This finding has been previously discussed by other authors who suggested that intrinsic adaptive mechanisms of glucose counter-regulation (e.g., glucagon and catecholamines release) are blunted in T1DM patients previously exposed to hypoglycemia, resulting in increased severity of hypoglycemic episodes.^46^^,^^47^ One plausible reason for the delayed NVC response may be an alteration in the function of potassium inwardly rectifying (Kir) channels, which are crucial for capillary-to-arteriole dilation transmission.^39^ Dysfunction of this vascular mechanism can cause delayed NVC without affecting the magnitude of the dilation. A necessary component for the functionality of both Kir and K-ATP-dependent channels is ATP production, which is altered in the context of STZ-induced hyperglycemia,^48^ suggesting a metabolic switch under hyperglycemic conditions. While NVC is delayed at 3 h after RH, the system’s functionality is restored after 24 h, indicating that between 3 and 24 h homeostasis is reinstated and the effect of non-severe hypoglycemic episodes on brain vasculature is short-lived at PA level. Further studies are needed to elucidate both the specific mechanisms related to the acute delay of NVC in the context of diabetes and the metabolic adaptation that allows the maintenance of vascular homeostasis during persistent hyperglycemia and RH. It is possible that longer durations of hypoglycemia or a higher frequency of recurrent episodes could induce dysfunction at the PA level. However, modeling prolonged periods of non-severe hypoglycemia is technically challenging using insulin-induced hypoglycemia, as extending the duration often leads to glucose levels that fall into the severe hypoglycemia range. Alternative approaches, such as the hypoglycemic clamp technique, would be more suitable for this purpose. On the other hand, increasing the number of hypoglycemic episodes disrupts the compensatory mechanisms that regulate glucose homeostasis. Therefore, a specific study incorporating hormonal monitoring and control of the counter-regulatory response would be required to accurately assess the impact of repeated hypoglycemic episodes on PA functionality.

### NVC in T1DM vs. T2DM: The putative role of comorbidities

Although T1DM and T2DM share the feature of glucose dysregulation, they are fundamentally distinct diseases with different pathophysiological mechanisms and clinical presentations. T1DM is primarily an autoimmune disorder characterized by the destruction of pancreatic β-cells, leading to absolute insulin deficiency. In contrast, T2DM is a heterogeneous metabolic disorder associated with insulin resistance, obesity, and a cluster of comorbidities such as hypertension, dyslipidemia, and cardiovascular disease. These differences raise an important question: How applicable are findings from studies on NVC in T2DM in the context of T1DM?

NVC impairments have been widely documented in T2DM, both in humans and animal models. Studies have shown that individuals with T2DM exhibit cerebrovascular dysfunction, including reduced cerebral blood flow and impaired vascular reactivity, which are exacerbated by the presence of comorbidities such as hypertension and atherosclerosis.^10^^,^^11^^,^^49^^,^^50^ In rodent models of T2DM, impaired NVC responses have been linked to endothelial dysfunction, oxidative stress, and chronic low-grade inflammation.^51^ However, since T2DM is often diagnosed alongside these metabolic and vascular complications, it is challenging to isolate the specific contribution of hyperglycemia to NVC deficits in models of T2DM. The STZ-induced model of hyperglycemia closely resembles T1DM and allows investigation of the direct impact of glucose dysregulation on NVC, minimizing the confounding metabolic factors associated with T2DM. Our findings suggest that after 8 weeks of sustained hyperglycemia, NVC remains largely preserved in PAs, indicating that the brain vasculature can adapt to chronic hyperglycemia without significant functional impairment. This aligns with previous reports showing that cerebral blood flow remains unchanged in T1DM models, even under conditions of acute hyperglycemia.^14^^,^^52^ However, other studies have reported decreased NVC in pial arteries of T1DM rats 16–18 weeks after diabetes induction, suggesting that vascular impairment may develop over a longer time course than the one used in our study.^12^^,^^13^ The discrepancy between our findings and previous studies may also be due to differences in species (mice vs. rats), vascular compartments analyzed (PAs vs. pial arteries), brain region analyzed (given that different brain regions are characterized by their unique patterns of NVC^53^), and the duration of hyperglycemia exposure. Determining whether NVC deficits eventually emerge in STZ-induced hyperglycemia and if specific vascular compartments exhibit differential vulnerability to metabolic stress are yet to be explored.

As a conclusion, our findings reveal that NVC at the PA level remains intact in a mouse model of STZ-induced hyperglycemia, as well as in hyperglycemic mice subjected to RH, preserving vascular tone and dilation capacity, indicating that this vascular compartment is highly resilient to glucose dysregulation. However, RH induces a transient delay in NVC response in diabetic mice, potentially reflecting a temporary vascular adaptation. Unlike T2DM, where comorbidities contribute to cerebrovascular dysfunction, sustained hyperglycemia alone does not impair NVC at the PA level. These findings underscore the need to differentiate between diabetes types in cerebrovascular research and the importance of further studies on metabolic adaptations in the diabetic brain vasculature.

### Limitations of the study

An important aspect not addressed in this study is the endocrine counter-regulatory response triggered during hypoglycemia. Under normal glucose homeostasis, a drop in circulating glucose stimulates glucagon release from pancreatic α-cells, which promotes hepatic glucose production through glycogenolysis and gluconeogenesis. This response is complemented by autonomic activation and adrenaline release, which further enhance hepatic glucose output and limit peripheral glucose utilization.^54^^,^^55^ In insulin-deficient states such as T1DM or STZ-induced hyperglycemia, the glucagon release can be impaired or delayed, increasing vulnerability to severe hypoglycemia. Repeated hypoglycemic episodes can progressively blunt these hormonal responses, leading to hypoglycemia-associated autonomic failure (HAAF). This condition influences both the magnitude of glucose reduction and its effects on the brain, thereby modulating the depth and duration of hypoglycemic episodes and increasing the risk of severe hypoglycemia.^47^^,^^56^^,^^57^ Studies distinguishing between mild-to-moderate and severe hypoglycemia or examining how these different degrees of glucose reduction differentially affect counter-regulatory hormonal responses under euglycemic and hyperglycemic conditions may provide deeper insight into the continuum of glycemic dysregulation. Our study pursued a targeted strategy, prioritizing the effects of reduced circulating glucose on NVC over a direct assessment of hormonal dynamics. Future studies quantifying insulin, glucagon, and catecholamines will be essential to elucidate the endocrine contribution to hypoglycemia-induced brain responses under both normoglycemic and STZ-induced hyperglycemic conditions.

This study has notable strengths, assessing the NVC response in awake mice using a longitudinal approach, reinforcing the validity of our findings. The study also provides an accessible platform for studying NVC in the somatosensory cortex that includes the codes and custom-designed pieces utilized and available at GitHub. A technical limitation of our study, however, is that our current awake setup makes the quantification of NVC at the capillary level quite unreliable due to movement artifacts inherent to awake imaging. Such analysis would be valuable to determine the specific effects of sustained hyperglycemia and RH on capillaries compared to PAs in future studies.

## Resource availability

### Lead contact

Further information and requests for resources and reagents should be directed to and will be fulfilled by the lead contact, Dr. Ricardo Mostany (rmostany@tulane.edu).

### Materials availability

This study did not generate new unique reagents.

### Data and code availability

All data reported in this paper are available from the lead contact upon request. All original code has been deposited at GitHub (https://github.com/mostanylab/NVC) and is publicly available as of the date of publication. DOIs are listed in the key resources table. Any additional information required to reanalyze the data reported in this paper is available from the lead contact upon request.

## Acknowledgments

This work was supported by awards from the 10.13039/100000002National Institutes of Health to R.M. and P.V.G.K. (R01NS114286 and 1R01AG074489) and to R.M. (1P01AG071746).

## Author contributions

I.F.U., P.V.G.K., A.Z., and R.M. designed the study; I.F.U., J.C.I., and C.M.D. performed the experiments; I.F.U., V.M.C.H., and H.S. analyzed the data; I.F.U. and R.M. wrote the manuscript. All authors have read and approved the final version of the manuscript.

## Declaration of interests

The authors declare no competing financial interests.

## STAR★Methods

### Key resources table

**Table:** 

REAGENT or RESOURCE	SOURCE	IDENTIFIER
**Chemicals, peptides, and recombinant proteins**		

Streptozotocin	Merck	Cat#S0130
Glucose	Merck	Cat#G7021
Humulin R	Eli Lilly Canada INC	NDC#0002-8215-01
Carprofen (Rimadyl)	Zoetis	NDC#54771-8461-3
Dexamethasone	VetOne	NDC#13985-037-02
Fluorescein isothiocyanate 70,000 KDa	Merck	Cat#90781

**Critical commercial assays**		

Insulin ELISA (mouse)	Crystal Chem	Cat#90080
Insulin ELISA (human)	Crystal Chem	Cat#90095

**Experimental models: Organisms/strains**		

Mouse: C57BL/6J	The Jackson Laboratory	Jax: 000664

**Software and algorithms**		

Neurovascular coupling quantification code	This paper. Deposited in Zenodo	https://doi.org/10.5281/zenodo.17541129
MATLAB	MathWorks	RRID: SCR_001622 https://www.mathworks.com/products/matlab.html
ScanImage 2015b	Pologruto et al.^53^	RRID: SCR_014307, https://www.mbfbioscience.com/products/scanimage/
GraphPad Prism	GraphPad Prism	RRID: SCR_002798 https://www.graphpad.com/

**Other**		

Glucometer Contour Next EZ	Ascensia Diabetes Care US INC	Cat#9647
Stainless steel bone screws	Protech Int.	00-90 X 1/16
Headbars	This paper. Deposited in Zenodo	https://doi.org/10.5281/zenodo.17541129
Headbar holders	This paper. Deposited in Zenodo	https://doi.org/10.5281/zenodo.17541129
Contemporary Ortho Jet Powder Dental Acrylic	Lang Dental Mfg. Co., Inc	Cat#1520BLK
Contemporary Ortho Jet Liquid Dental Acrylic	Lang Dental Mfg. Co., Inc	Cat#1304CLR
Pneumatic drill	Midwest Tradition	Cat#393-8805
Stereotactic frame	Stoelting	Cat#51600
Picospritzer III	Parker Hannifin Corp.	P/N 052-0500-900
PICMA multilayer Piezo bender actuator	Physik Instrumente	Cat#PL140.11
Intrinsic Optical Signaling camera Pantera 1M60	Dalsa	Cat#DS-1A-01M30
Intrinsic Optical Signaling frame grabber 64 Xcelera-CL	Dalsa	Cat#OR-X1C0-XLB00

### Experimental model and study participant details

#### Animals

Both female and male young (2-3 months-old at the onset of the experiment) wild type (C57BL6/J) mice were used for the studies. Mice were maintained under controlled temperature and light conditions (12-hour light/dark cycle). Food and water were provided *ad libitum*. All procedures were approved by the Tulane University Institutional Care and Use Committee (protocol number 1721) and were performed in accordance with the NIH office of Laboratory Animal Welfare’s Public Health Service Policy on Humane Care and Use of Laboratory Animals and Guide for the Care and Use of Laboratory Animals.

### Method details

#### Experimental design

We conducted a longitudinal study to evaluate the NVC response to whisker stimulation in awake mice of a streptozotozin- (STZ) induced hyperglycemia model (Figure 1A). To achieve this, we implanted a cranial window to optically access the primary somatosensory barrel field cortex (S1BF; Figures 1B and 1C). After a two-week recovery period, the mice were habituated to the awake imaging setup of the two-photon microscope, and immediately following habituation, we performed the first NVC evaluation in the penetrating arterioles of the S1BF to establish a baseline (BL) response. Next, we induced experimental diabetes in a group of mice using STZ, while maintaining a control group of non-induced mice. Eight weeks after diabetes induction, mice were re-habituated to the awake imaging setup, and we re-evaluated the NVC in the same penetrating arterioles to determine the effect of sustained hyperglycemia (Pre-RH). Then, mice underwent RH and we assessed NVC of the same PAs at 3 hours (RH-3h) and 24 hours (RH-24) after the last episode of the RH protocol.

#### Blood glucose measurements

Glucose levels were measured after a 5-hour fasting period. Blood collection was done by pricking the lateral tail vein with a 21G needle and applying gentle pressure to extract a drop of blood. Glucose levels were measured with a handheld glucometer (Contour Next EZ, Bayer). Blood draws were performed before diabetes induction, as well as 2 and 8 weeks later, following a 5-hour fasting period to determine fasting blood glucose levels. Additionally, blood draws were performed every 15 minutes during hypoglycemic episodes to ensure blood glucose levels were maintained within the target range.

#### Hyperglycemia induction

Sustained hyperglycemia was induced following a 5-day protocol consisting of daily low-STZ dose injections, as previously described.^33^ Each day mice were fasted for 5 hours before receiving an intraperitoneal injection of STZ (50 mg/kg) dissolved in 100 mM sodium citrate buffer, pH 4.6. Nondiabetic control mice received injections of the citrate buffer alone. After each injection, the mice were monitored for 3 hours before being returned to their cages. Blood glucose levels were assessed before the first STZ dose, and again at 2 weeks and 8 weeks after the final STZ administration. Mice were considered hyperglycemic if fasting glucose levels exceeded 200 mg/dL at the two-week time point.

#### Glucose tolerance tests (GTT)

Mice were fasted for 6 h before the GTT assessment. For this, mice were moved to individual clean cages with no food but unrestricted access to water. Blood samples were collected from the tip of the tail vein before and after (15, 30, 60, 90, and 120 min) a single intraperitoneal injection of 20% glucose (2 g/kg body weight). Blood glucose levels were measured using a glucometer (OneTouch Verio Flex). Glucose levels across time points were used to calculate the area under de curve (AUC), and the initial time point before the glucose injection was used as the fasting glucose levels.

#### Recurrent hypoglycemia protocol

Five episodes of mild-to-moderate hypoglycemia (45-70 mg/dL of blood glucose) were induced over five consecutive days by administering insulin (1-4 UI/kg, Humulin R, Lily) once per day after 4 hours of fasting. The amount of injected insulin was calculated based on the initial glucose levels and following the formula: Insulin dose (UI) = 0.75 x body weight (kg) x fasting blood glucose (mg/dL)/100. This formula was optimized to maintain sustained hypoglycemia within a blood glucose range of 45-70 mg/dl. Glucose levels were monitored every 15 minutes after the insulin injection. After one hour of hypoglycemia, mice received an intraperitoneal injection of glucose (1.5 g/kg) to rescue them from the hypoglycemic episode.

#### Serum insulin levels

To validate the successful induction of a chronic hyperglycemic state, we measured insulin levels in serum from control and STZ-treated mice eight weeks after STZ administration. Blood samples were obtained from the heart during animal euthanasia, and were centrifugated at 10,000xg for 15 min. Serum was obtained and stored at -80°C until use. Insulin levels were measured with an ELISA assay (Crystal Chem), following manufacturer’s instructions.

#### Plasma Humulin-R levels

To determine whether any injected insulin remained in the system 3 hours after recovery from the RH episode, we used an ELISA kit specifically for human insulin, taking advantage of the fact that the injected Humulin-R is human insulin. Humulin-R levels were measured in plasma obtained from diabetic mice at various time points after injection. Briefly, diabetic mice were injected with 0, 1.5, 2, or 4 IU/kg of Humulin-R and were sacrificed either 1-hour post-injection (corresponding to the onset of the hypoglycemic phase) or 5 hours post-injection (corresponding to 3 hours after the RH episode). Blood was collected from the cheek and placed in anticoagulant solution (0.5 M EDTA) in a 1:10 ratio (anticoagulant:blood). Blood was then centrifuged at 10,000xg for 10 minutes, and the obtained plasma was stored at -80°C for further use. Blood Humulin-R levels were measured using a specific ELISA kit (Crystal Chem) designed to detect human insulin with minimal cross-reactivity to mouse or rat insulin and following manufacturer’s instructions.

#### Cranial window surgery

For the cranial window procedure,^58^^,^^59^^,^^60^ mice were anesthetized with isoflurane (5.0% for induction, 1.4-1.7% for maintenance). Dexamethasone (0.2 mg/kg; VetOne) and carprofen (5.0 mg/kg, Zoetis, Inc) were subcutaneously injected before the surgery to prevent brain swelling and inflammation. Mice were placed on a stereotaxic frame (Stoelting) to perform the surgical procedure. After bone exposure, two 00-96 x 1/16” screws (Protech Int.) were implanted on the skull of the contralateral hemisphere (frontal and parietal bone) to strengthen and provide stability to the acrylic headcap that embeds the head bar utilized to secure the animals to the microscope stage during awake imaging. A 4 mm craniotomy was performed with a pneumatic dental drill (Midwest Tradition) over the S1BF (AP: -1.95, ML: +3.0), keeping the dura intact. A 5 mm coverslip (#1; Electron Microscopy Sciences) was placed over the exposed brain and fixed to the skull with ethyl-cyanoacrylate glue. A custom designed head bar (Figure 1B inset) was placed over the cranial window and secured with dental acrylic (Lang Dental Mfg. Co., Inc) to the skull. The animal recovered for 2-3 weeks before starting with the rig habituation protocol.

#### Intrinsic optical signal imaging

Intrinsic optical signal (IOS) was performed in the time between cranial window surgery recovery and the first session of NVC imaging, i.e., baseline time point, to identify the location of the S1BF region of the parietal cortex in the cranial window. IOS imaging was performed as we have previously described.^61^^,^^62^ Mice were anesthetized (5% isoflurane for induction, <1% for maintenance) and secured to a custom-built IOS rig using the titanium head bar. An image of the surface’s vasculature was obtained for reference under green light (535 nm). Whiskers of the contralateral side to the cranial window were gently applied to a piezo bender actuator (Physik Instrumente) using dental wax. Then, intrinsic optical signals at 300 ± 50 μm deep were recorded under red light (630 nm) with a fast camera (Pantera 1M60; Dalsa), frame grabber (64 Xcelera-CL PX4; Dalsa), using a custom written MATLAB (MathWorks) routine. The imaging session consisted of 30 trials of whisker stimulation for 1.5 seconds in the rostro-caudal direction at 10 Hz with 20 second breaks. The response signals for each stimulation were normalized to their baseline signal and summed up to obtain the activity map. The map of activity was overlaid with the vasculature’s to identify the activated cortical area that corresponds to S1BF. Once the functional map of this cortical area was reliably and consistently identified in a subset of animals (Figure 1B), the region was generalized to the rest of mice, since the position of the cranial window does not vary between animals.

#### Habituation

Because neurovascular coupling was assessed in awake mice to avoid the dampening effect of anesthesia on functional hyperemia, mice needed to be habituated to the imaging setup before the imaging sessions. Mice were habituated for 5 days before the first imaging session (Baseline, or BL) and again before the second imaging of NVC (Pre-RH) given the long time interval, 8 weeks, between these two sessions (Figure 1A). For the habituation (H), mice were lightly anesthetized with isoflurane (4% isoflurane for 30 seconds) and placed on the custom-designed imaging platform by securing the head bar to the head bar holders. The body of the animal rested inside a metallic cylinder. The animal was allowed to fully wake up on the platform. The habituation consisted of increased restraining times across five consecutive days as follows: 20, 45, 60, and 2 x 90 minutes. On days 4 and 5 the restraining time was combined with 25 min of whisker stimulation using air puffs generated with a Picospritzer III (4Hz, 5 s, 20 psi, 30 s/cycle) to get the animals used to the whisker stimulation paradigm.

#### Two-photon laser scanning microscopy of neurovascular coupling

Two-photon laser scanning microscopy (2PLSM) imaging was performed in a custom-built microscope equipped with a Ti:sapphire laser (Chameleon Ultra II; Coherent) tuned to 910 nm, a 16x 0.8 NA water-immersion and long working distance objective (Nikon), and ScanImage software^63^ written in MATLAB. Mice were lightly and transiently anesthetized with isoflurane (4% isoflurane for 30 seconds), and a bolus of 100 μL of fluorescein isothiocyanate-dextran 70 kDa (FITC, 5% w/v in sterile saline) was injected retro-orbitally to label the blood serum and visualize the vasculature.^64^^,^^65^ Immediately after the injection, mice were secured to the custom-designed imaging platform and positioned directly under the microscope objective. Mice were allowed to fully recover from anesthesia for 15-30 minutes before the start of the imaging session. Cross sections of PAs were obtained 50 ± 20 μm deep from the pial arteries and were imaged at 9.45 Hz for a total duration of 25 s per trial. For each trial, the whisker stimulation was done using air puffs (4Hz, 20 psi, 5 s) and initiated 10 seconds after the onset of the image acquisition. An Arduino-based custom-built I/O controller was used to synchronize the imaging acquisition and the onset of the stimulation. Ten stimulation trials were performed for each PA and averaged to minimize variability due to artifacts associated with the imaging of awake mice. Two to six PAs per mouse were imaged in the S1BF. PAs were imaged longitudinally across time points and their responses were normalized to their basal NVC response (BL). A representative video of the methodology can be found in Video S1.

Video S1. Representative video of the acquisition of brain vascular dynamics in awake mice using two-photon microscopy

### Quantification and statistical analysis

#### Image analysis quantification of NVC responses

The changes in PA diameter for each stimulation trial were quantified using a custom-written routine in MATLAB. Briefly, image intensity of the cross-section of the PA was adjusted, binarized, and the centroid of the resulting mask was calculated and used to measure the minimum diameter of the PA at every frame recorded. Basal diameter was measured from second 3 to the onset of the whisker stimulation (at second 10), and the average basal diameter for each trial was used to normalize the stimulation-induced changes in diameter (being basal diameter 100%). The traces from the 10 trials per PA were averaged to reduce variability from artifacts related to awake imaging. NVC diameter response was evaluated between seconds 10 and 15, corresponding to the stimulation time. For each PA and imaging session, the maximum amplitude of the PA during the response to the stimulation (Amplitude), the times to reach the maximum amplitude (t_Max_) and 50% of the maximum amplitude (t_Amp50%_), as well as the area under the curve during the stimulation period (AUC) were obtained. These parameters were compared for the same PAs across time points in the study, and the percentage of change between the different time points was calculated to evaluate the effects of hyper- and hypoglycemia on the NVC response.

#### Statistical analysis

Statistical analysis was performed in GraphPad Prism. Specific tests that were used to test significance in each plot can be found in figure legends. The statistically significant results are described in Results. For the analysis of NVC responses, a total of 2-6 PAs were collected per mouse. To avoid bias and appropriately weigh the contribution of mice with different numbers of PAs, all data were analyzed using either *nested* one- or two-way ANOVA, followed by *Tukey's post hoc* analysis for multiple comparisons. Data are presented as mean ± standard deviation (SD), with significance determined at *p* < 0.05. Representation of significance is set as ∗ *p* < 0.05, ∗∗ *p* < 0.01, ∗∗∗ *p* < 0.001, and ∗∗∗∗ *p* < 0.0001.
